# Dysregulation of the Ubiquitin Proteasome System in Human Malignancies: A Window for Therapeutic Intervention

**DOI:** 10.3390/cancers13071513

**Published:** 2021-03-25

**Authors:** Chee Wai Fhu, Azhar Ali

**Affiliations:** Cancer Science Institute of Singapore, National University of Singapore, Singapore 117599, Singapore; csifcw@nus.edu.sg

**Keywords:** ubiquitin proteasome system, dysregulation, chemoresistance, cancer, therapy, inhibitors

## Abstract

**Simple Summary:**

The ubiquitin proteasome system (UPS) governs the non-lysosomal degradation of oxidized, damaged, or misfolded proteins in eukaryotic cells. Dysregulation of the UPS results in loss of ability to maintain protein quality through proteolysis, and is closely related to the development of various malignancies and tumorigenesis. Here, we provide a comprehensive general overview on the regulation and roles of UPS and discuss the mechanisms linking dysregulated UPS to human malignancies. Inhibitors developed against components of the UPS, which include U.S. Food and Drug Administration FDA-approved and those currently undergoing clinical trials, are also presented in this review.

**Abstract:**

The ubiquitin proteasome system (UPS) governs the non-lysosomal degradation of oxidized, damaged, or misfolded proteins in eukaryotic cells. This process is tightly regulated through the activation and transfer of polyubiquitin chains to target proteins which are then recognized and degraded by the 26S proteasome complex. The role of UPS is crucial in regulating protein levels through degradation to maintain fundamental cellular processes such as growth, division, signal transduction, and stress response. Dysregulation of the UPS, resulting in loss of ability to maintain protein quality through proteolysis, is closely related to the development of various malignancies and tumorigenesis. Here, we provide a comprehensive general overview on the regulation and roles of UPS and discuss functional links of dysregulated UPS in human malignancies. Inhibitors developed against components of the UPS, which include U.S. Food and Drug Administration FDA-approved and those currently undergoing clinical trials, are also presented in this review.

## 1. The Ubiquitin Proteasome System

The ubiquitin proteasome system (UPS) is essential for the regulation of protein homeostasis and control of eukaryotic cellular processes including cell cycle progression, stress response, signal transduction, and transcriptional activation [1,2]. UPS controls the degradation of approximately 80% of intracellular proteins which are oxidized, damaged, or misfolded in eukaryotic cells [3]. Though the UPS and autophagy are both important systems of degradation of proteins, the sizes of substrates critically influence the choice of degradation pathway [4]. The UPS typically degrades single unfolded polypeptides, whereas autophagy deals with larger cytosolic complexes, cellular aggregates, and organelles.

**Figure 1:**
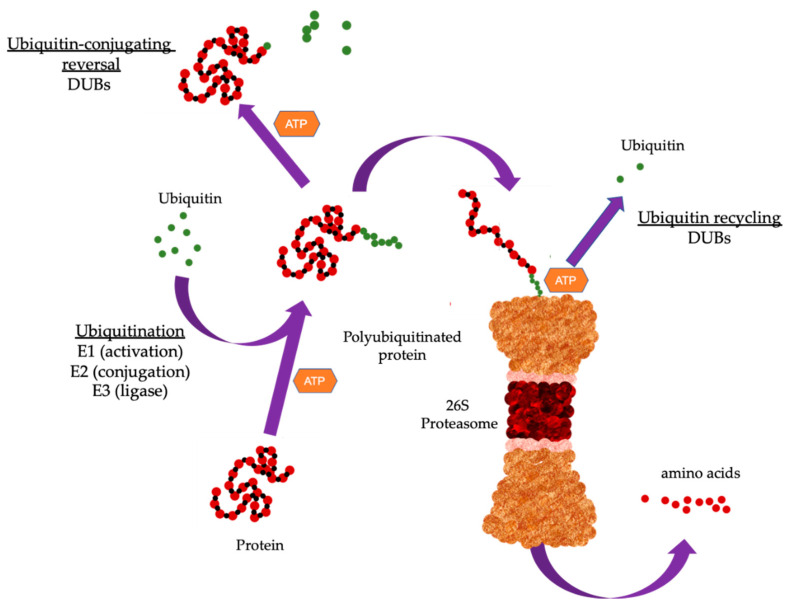
Overview of the ubiquitin proteasome system (UPS). The UPS cascade. Substrate protein is ubiquitinated through the sequential action of three enzymes. E1 binds to activated ubiquitin and is transferred to the ubiquitin-conjugating enzyme (E2). The E2 carries the activated ubiquitin to ubiquitin ligase (E3), which then facilitates the transfer of ubiquitin from E2 to a lysine residue in the target protein. Proteins can be modified with a single mono-ubiquitin molecule, or with ubiquitin chains of different lengths and linkage types. Substrate proteins modified with specific chains are recognized and subsequently degraded by the 26S proteasome. Deubiquitinating enzymes (DUBs) remove ubiquitin from substrate proteins by removing mono-ubiquitination or by trimming or removing the ubiquitin chain. Typically, poly-ubiquitination has been associated with protein clearance through proteasomal degradation while mono-ubiquitination which involves the addition of a single ubiquitin moiety to the substrate protein affects cellular processes.

Degradation of targeted proteins involves a tightly coordinated process where ubiquitin is covalently attached to the substrate protein through the sequential action of three enzymes. Ubiquitin is a small protein comprising 76 amino acids found in all eukaryotic cells [5]. The energy derived from ATP hydrolysis initiates the activation of ubiquitin activating enzyme (E1) allowing the formation of thioester bond between E1 and ubiquitin. This is followed by transfer of ubiquitin from E1 to ubiquitin-conjugating enzyme (E2), forming a thioester bond similar to that of E1. The third final step involves the covalent attachment of ubiquitin to lysine residues of target protein, catalyzed by ubiquitin ligase (E3) [6]. The 26S proteasome complex comprises a core 20S proteasome and one or two units of the regulatory 19S proteasome (Figure 1). Once a target protein has been modified with a polyubiquitin chain, it is recognized by the 19S proteosome which removes the polyubiquitin chain and the protein is then unfolded and translocated into the 20S proteasome where it is degraded into short peptides [7]. While polyubiquitination has been associated with protein clearance through proteasomal degradation, mono-ubiquitination, which involves the addition of a single ubiquitin moiety to the substrate protein, is shown to affect a range of cellular processes including kinase activity, epigenetic regulation, protein translocation, and DNA damage signaling [8,9].

Ubiquitin contains seven important lysine residues which can be ubiquitinated (K6, K11, K27, K33, K48, and K63) and can form polyubiquitin chains. The two best characterized ubiquitin linkages are K48 and K63 where K48 polyubiquitination targets proteins for degradation by the 26S proteasome complex [10] and K63 participates in DNA damage signaling and recruits DNA repair proteins to damage sites [11]. Protein ubiquitination can be reversed through the removal of ubiquitin from target proteins by deubiquitinating enzymes (DUBs), and this rescues protein destined for degradation. DUBs have also been implicated in the maturation, recycling, and editing of ubiquitin [12,13,14]. Further, chain configuration and linkage can endow ubiquitin with additional roles through the formation of more complex topologies with unknown activities [15]. Dysregulation or abnormal UPS function is frequently seen in various human malignancies and this identifies the aberrant components of the UPS as potential drug targets [16,17]. This review endeavors to present recent literature on the functional roles of UPS in human cancers. We cover how the dysregulation of UPS components may function either as an oncogene or tumor suppressor and affects cellular signaling in tumors. Further, we present current small inhibitors against the UPS and highlight issues that has severely restricted its development.

Increasing evidence demonstrate ubiquitin enzymes are important in carcinogenesis. Though there are numerous cancer-related studies on these enzymes, a large majority primarily focuses on E3 ligases. Studies on E1-activating enzymes have been largely used to identify potential targets in UPS inhibition in cancer while studies on E2-conjugated enzymes revealed their involvement in cell cycle progression, DNA repair, and regulation of oncogenic signaling pathways during tumorigenesis [18,19]. Further E2 enzymes are often found upregulated and highly correlated with poor prognosis in various malignancies including the pancreas, lung, breast, skin, and thyroid [20]. Currently, eight E1s, > 40 E2s, and > 600 E3s have been identified in the human proteome [21].

## 2. Ubiquitin-Conjugating Enzymes

The ubiquitin-conjugating E2 family comprises > 40 members, and modulates protein stability and ubiquitination through the conjugation of ubiquitin to target proteins [22]. E2-conjugating enzymes are also found dysregulated in cancers and reported to be potent mediators contributing towards multiple tumorigenic processes including migration/invasion, proliferation, drug resistance, radiation resistance, cell cycle, apoptosis, and stimulation of oncogenic pathways (Table 1). We present selected E2 members with roles in cancer below.

### 2.1. UBE2C

Ubiquitin-conjugating enzyme E2 C (UBE2C) is expressed at critical points during cell cycle progression, which strongly relates to its role in cell cycle regulation [23]. UBE2C overexpression is associated with various malignancies, and shown to play a role in cancer cell growth and invasive properties [24,25]. UBE2C overexpression has been correlated with poor prognosis in cancers of the stomach, colorectal, thyroid, breast, and pancreas [23,25,26,27,28]. UBE2C promotes drug resistance in breast cancer, as the UBE2C blockade enhances tumor cell sensitivity to doxorubicin [29]. This observation suggests that UBE2C expression can serve as a marker for doxorubicin sensitivity in breast cancer patients. Similarly, silencing of UBE2C enhances sensitivity to cytotoxic drugs in hepatocellular cancer [30]. Upregulated UBE2C plays an oncogenic role in human intestinal-type gastric cancer, where UBE2C silencing in these cells disrupts the cell cycle at the G2/M stage. In gastric cancer, UBE2C overexpression induces chromosomal instability and perturbs the cell cycle [23]. In non-small cell lung cancer (NSCLC), elevated UBE2C expression has been reported in cisplatin-resistant NSCLC cells, which correlates with high proliferation and invasion. Further, UBE2C silencing in high UBE2C expressing NSCLC cells induced the upregulation of E-cadherin with concomitant downregulation of vimentin. UBE2C can also bind to the 5′-UTR of drug resistance-related genes such as ERCC1 and ABCG2. Collectively, these data suggest that targeting UBE2C can suppress cisplatin resistance and reverse epithelial to mesenchymal transition (EMT) in NSCLC [31].

**Table 1:** Summary of the functions of E2 and E3 enzymes in human cancers described in this review.

Family	Name	Role	Cancer Type	Function	Test Model	Reference
*E2*	UBE2C	Oncogene	Gastric	Chromosomal stability, Proliferation, Migration, Invasion	In vitro, In vivo	[23]
		Oncogene	Colon	Cell cycle, Proloferation	In vitro	[24]
		Oncogene	Colorectal	Proliferation, Invasion	In vitro	[25]
		Oncogene	Thyroid	Proliferation	In vitro	[26]
		Oncogene	Breast	Proliferation, Drug resistance, Radiation resistance	In vitro	[29]
		Oncogene	Liver	Proliferation, Drug resistance, Migration, Invasion	In vitro	[30]
		Oncogene	Non-small cell lung	Drug resistance	In vitro	[31]
	UBE2Q1	Oncogene	Colorectal	Proliferation		[32]
		Oncogene	Liver	p53 signaling, Cell cycle	In vitro	[33]
		Oncogene	Breast	p53 signaling	In vitro	[34]
	UBE2S	Oncogene	Endometrial	SOX6/β-catenin signaling, Proliferation	In vitro	[35]
		Oncogene	Lung adenocarcinoma	Proliferation, p53 signaling, Apoptosis	In vitro	[36]
		Oncogene	Liver	p53 signaling, Cell cycle	In vitro	[37]
*E3*	FBW7	Tumor suppressor	Burkitt’s lymphoma	c-Myc signaling	In vitro	[38,39]
		Tumor suppressor	Chronic myelogenous leukemia	c-Myc signaling	In vitro, In vivo	[40]
		Lipogenesis	Lung, Melanoma, Thyroid, Cervical	mTORC2/SREBP1 signaling	In vitro	[41]
		Tumor suppressor	T cell leukemia	Notch signaling	In vitro, In vivo	[42]
		Tumor suppressor	Colorectal	c-Myc signaling, Cell cycle	In vitro	[43]
		Tumor suppressor	Esophageal squamous cell	c-Myc signaling	In vitro	[44]
		Tumor suppressor	Colorectal, Cervical, Ovarian, Non-small cell lung	Apoptosis (via Mcl1)	In vitro	[45]
	MDM2	Oncogene	Neuroblastoma	p53 signaling	In vitro, In vivo	[46]
		Oncogene	Cervical	Cell cycle, Apoptosis	In vitro	[47]
		Oncogene	Liver	Metastasis, Drug response	In vitro, In vivo	[48]
	Cdc20	Oncogene	Breast	Metastasis, Drug response	In vitro	[49]
	Cdh1	Tumor suppressor	Breast	Src signaling	In vitro	[50]
	β-TRCP	Tumor suppressor	Breast, Prostate	MTSS1 signaling	In vitro	[51]
		Oncogene	Lung	FOXN2	In vitro, In vivo	[52]
		Tumor suppressor	Papillary thyroid	VEGFR2 signaling	In vitro, In vivo	[53]
	E6AP	Oncogene	Prostate	Radiation response	In vitro	[54]
		Oncogene	Prostate	p27 signaling	In vitro, In vivo	[55]
		Oncogene	Prostate	Metastasis	In vitro, In vivo	[56]

### 2.2. UBE2Q1

Ubiquitin-conjugating enzyme E2 Q1 (UBE2Q1) dysregulation has been observed in several cancers including acute lymphocytic leukemia (ALL), breast, hepatocellular, and colorectal [32]. Higher UBE2Q1 levels have been detected in hepatocellular carcinoma (HCC) tumors compared to adjacent normal tissues. Further, elevated UBE2Q1 is significantly correlated with advanced stages and confers poor prognosis in HCC. Silencing of UBE2Q1 by siRNAs reduces proliferation, induces cell cycle arrest, and upregulates tumor suppressor protein 53 (p53) and cyclin-dependent kinase inhibitor 1 (p21) in HCC cells [33]. In breast cancer cells, UBE2Q1 overexpression downregulates p53 and increases cell resistance to apoptosis, indicating that UBE2Q1 offers a protective role in tumor cells through ubiquitination and proteasome degradation of p53 [34].

### 2.3. UBE2S

Ubiquitin-conjugating enzyme E2 S (UBE2S) overexpression enhances migration and growth of endometrial cancer cells, while silencing it can reverse these effects. Upregulation of UBE2S in these cells can induce nuclear translocation of β-Catenin through SOX6 suppression, and cyclin D1 and c-Myc up regulation [35]. UBE2S increases β-catenin stability by inhibiting its degradation. The β-catenin blockade inhibits UBE2S-induced cancer cell expansion, suggesting that components of the UBE2S-SOX6/β-catenin axis are possible therapeutic targets of endometrial cancer. Overexpression of UBE2S has also been observed in lung cancer cell lines where UBE2S silencing reduces colony formation and increased apoptosis [36]. UBE2S is also reported to be a prognostic factor of hepatocellular carcinoma, and targeting UBE2S in high UBE2S-expressing cells restricts the proliferation and migration of tumor cells where increased expression of p53, p21, and cyclin D1 is seen after UBE2S suppression [37].

## 3. Ubiquitin Ligases

E3 ubiquitin ligases are a large family of enzymes that promote ubiquitin transfer to proteins or polyubiquitin chains [21]. E3 ligases play an important role in the ubiquitin-mediated proteolytic cascade and are classified into four main classes, according to their domain structure and substrate recognition. The four E3 classes are the homologous to the E3 ubiquitin ligase E6-associated protein (E6AP) C-terminus (HECT), really interesting new gene (RING)-finger, U-box, and plant homeodomain (PHD)-finger. Depending on the substrate targets, E3 ligases can function either as a tumor suppressor or oncogene and can participate in various cellular processes including cell cycle, apoptosis, drug response, metastasis, radiation response, and oncogenic signaling (Table 1). We present selected E3 ligases with roles in cancer in the section below.

### 3.1. FBW7

F-box and WD repeat domain-containing 7 (FBW7) is a substrate recognition component of the Skp, Cullin, F-box (SCF) complex that regulates multiple pro-oncogenic pathways including c-Myc, Cyclin E, mTOR, and Notch [57,58,59]. FBW7 regulates the proteasomal degradation of c-Myc through phosphorylation of Thr58 and Ser62 residues, and mutation occurring at the Thr58 residue can promote Burkitt’s lymphoma progression [38,39]. In chronic myelogenous leukemia (CML), FBW7 deletion enhances c-Myc expression and can induce p53-dependent apoptosis in human leukemia-initiating cells [40]. FBW7 is shown to negatively regulate lipogenesis where the inhibition of FBW7 suppressor, mTORC2, stabilizes sterol regulatory element-binding protein 1 (SREBP1). FBW7 expression is shown to decelerate tumor progression in lung, thyroid, melanoma, and cervical cancer [41]. Further, tumor suppressor’s function of FBW7 is demonstrated where it suppresses Notch signaling and inhibits adult T-cell leukemia lymphoma progression [42]. More importantly, reduced FBW7 has been reported in various malignancies including hepatocellular, colorectal, and esophageal squamous cell carcinoma [43,44,60]. Though numerous studies have shown a protective role of FBW7 in cancer, there is controversy if FBW7 inhibition promotes or inhibits cancer as its impairment in cancer cells can induce chemoresistance by stabilizing oncogenic proteins [45]. This controversy can be attributed to the presence of FBW7 mutations which occur in ~10% of human cancers and the protein substrate which FBW7 is associated with. Currently, more than 20 substrates of FBW7 have been identified [61,62].

### 3.2. MDM2

Under normal physiological conditions, tumor suppressor protein 53 (p53) phosphorylation restrains mouse double minute 2 (MDM2)–p53 binding and this induces p53 overexpression in cells. In tumor cells, MDM2 can polyubiquitinate p53, leading to its proteasomal degradation, which is a major pathway altered in cancers [63,64]. MDM2 upregulation has been reported in various cancers, which renders it an attractive drug target for cancer therapy [46,47,48,65]. However, p53 mutations are frequently observed in tumors. These mutants are shown to bind to MDM2 with higher avidity than their wild-type counterpart, thus rendering them resistant to MDM2 inhibitors [66,67]. More importantly, MDM2 has been shown to be functionally linked with the regulation of cancer metabolism in animal models. In the mouse model, p53-independent roles of MDM2 have been reported where they mediate serine/glycine metabolism, which is an important oncogenic signaling in MDM2 overexpressing tumors [68].

### 3.3. Cdc20 and Cdh1

The anaphase promoting complex/cyclosome (APC/C) has two co-activators: Cdc20 and Cdh1. Cdc20 mRNA levels are elevated and are positively correlated with tumor size in breast cancer patients, suggesting its prognostic value [69]. Further, Cdc20 inhibition prevents breast cancer cell line migration and Cdc20 overexpression accelerates metastases of cancer cells in vitro [49]. Cdh1 is found dysregulated in breast cancer and melanoma, and triggers genome instability and weakens DNA damage response [50]. Cdh1 is also shown to delay G1 to S transition in cell cycle regulation, suggesting its role in tumorigenesis [70].

### 3.4. βTrCP

Beta-transducin repeats-containing protein (βTrCP) is overexpressed in breast and prostate cancer and the underlying molecular mechanism is that βTrCP promotes tumorigenesis by promoting the degradation of metastasis suppressor 1 (MTSS1) protein by UPS. Blocking MTSS1 degradation is thus a potential treatment for aggressive breast and prostate cancer [51]. βTrCP regulates the Forkhead box transcription factor (FOXN2) ubiquitination, and affects cell proliferation and radiosensitivity in lung cancer [52]. Further, βTRCP is shown to stimulate vascular endothelial growth factor receptor 2 (VEGFR2) ubiquitination and degradation, which inhibit angiogenesis and migration of papillary thyroid cancer cells [53]. These lines of evidence indicate that roles of βTRCP in cancer regulation are context-dependent.

### 3.5. E6AP

E6AP ubiquitin-protein ligase (E6AP) is recruited by the human papilloma virus E6 protein to promote p53 degradation, which is a key step in human papilloma virus-induced cancers [71]. E6AP is shown to slow down the growth of prostate cancer cell lines, and induces cell senescence in vivo. E6AP knockdown is also shown to increase sensitivity of cells to radiation-induced death [54]. In a separate study, E6AP was reported to inhibit the cell cycle regulator protein 27 (p27), acting through the E2F1-dependent pathway in prostate cancer [55]. Further, E6AP can suppress the metastasis suppressor n-Myc downstream regulated 1 (NDRG1) in mesenchymal phenotypes of prostate cancer where stabilization of NDRG1, via pharmacological inhibition of E6AP, has been proposed for prostate cancer therapy [56].

## 4. Deubiquitinases (DUBs)

DUBs rescue ubiquitin chains before the substrate protein is degraded by the 26S proteasome. Unbound ubiquitin chains are then processed by other DUBs to restore the free cellular ubiquitin pool. DUB genes are classified into two main groups– cysteine proteases and zinc metalloproteases [13]. DUBs can be categorized into five subclasses: Ubiquitin-specific proteases (USPs), ovarian tumor proteases (OTUs), Machado–Joseph domain proteases (MJDs), ubiquitin C-terminal hydrolases (UCHs), and Jab1/Mov34/Mpr1 Pad1 N-terminal+ (MPN+) (JAMM) domain metalloproteases [72]. Post-translational modification of proteins by ubiquitination affects the targeted protein in several ways: (i) It determines if the ubiquitinated proteins remain in the cell or to be degraded by the proteasome, (ii) it affects the cellular localization of proteins, (iii) it determines protein activity or inactivity, and (iv) it facilitates protein–protein interactions [73,74,75].

Currently, about >100 DUB genes have been identified where the biological functions for the majority are still unknown [13,14]. USPs are, by far, the largest class of DUBs, comprising ~60 human proteases with most containing several domains apart from the catalytic domain. Conserved sequences among these proteases are restricted to the catalytic domain which is designated by the catalytic motif containing Cys, His, and Asp (or Asn) residues. Conserved catalytic domains are thought to be important for substrate specificity, catalytic activity regulation, and mediating protein–protein interaction to each USP. Dysregulated DUBs in cancer are hence potential drug targets. The challenge in the development of drugs is the difficulty in designing a specific inhibitor for a single DUB. 3D crystallography structures of catalytic domains of USP2, USP7, USP8, and USP14 reveal a remarkable structural conservation of their active site shared among these enzymes, thus providing evidence that the development of inhibitors may prove to be challenging [76,77,78,79]. Further, the crystal structures show that the catalytic domains are in inactive conformation prior ubiquitin binding suggesting an alternative target for intervention. Apart from deubiquitination, DUBs have been shown to modulate cellular processes in human malignancies including DNA damage response, oncogenic signaling cascades, drug resistance, apoptosis, cell cycle, immunomodulation, and invasion/migration (Table 2). We will discuss several selected DUBs with roles in cancer below.

**Table 2:** Summary of the functions of DUB enzymes described in this review.

Name	Role	Cancer Type	Function	Test Model	Reference
*BAP1*	Tumor suppressor	Lung, Osteosarcoma, Colon	DNA double-strand repair	In vitro	[80,81,82]
	Tumor suppressor	Renal	Ferroptosis signaling	In vitro	[83]
*USP7*	Oncogene	Cervical	Self-renewal; Foxp3 signaling	In vitro	[84]
	Oncogene	Non-small cell lung	Immune Response; Foxp3 signaling	In vitro	[85]
	Oncogene	Lung	p53 signaling	In vitro, in vivo	[86]
*USP22*	Oncogene	Lung	Cell Cycle	In vitro	[87]
	Oncogene	Lung adenocarcinoma	EGFR-TKI resistance	In vitro, in vivo	[88]
	Oncogene	Colon	CCNB1 signaling	In vitro, in vivo	[89]
	Oncogene	Glioblastoma	KDM1A signaling	In vitro, in vivo	[90]
*UCHL1*	Oncogene	Breast	Drug resistance; Invasion/migration	In vitro	[91]
*Ataxin 3*	Oncogene	Breast, Osteosarcoma, Cervical, Colorectal	DNA	In vitro	[92]
	Oncogene	Testicular	mTOR/Akt signaling	In vitro	[93]
*PSMD11*	Oncogene	Cervical. Osteosarcoma	DNA damage response	In vitro	[94]
	Oncogene	Lung, Prostate, Colorectal, Breast, Cervix	Cell cycle	In vitro	[95]
	Oncogene	Liver	E2F1 signaling	In vitro, in vivo	[96]
*A20*	Tumor suppressor	Colorectal	Apoptosis signaling	In vitro	[97]
	Tumor suppressor	Diffuse large B-cell lymphoma	NF-***κ***β signaling	In vitro	[98]
	Tumor suppressor	Sarcoma	NF-***κ***β signaling	In vitro	[99]

### 4.1. BAP1

BRCA1-associated protein 1 (BAP1), a ubiquitin carboxy-terminal hydrolase (UCH), was identified in 1998 as a nuclear protein that bound to the RING-finger domain of the BRCA1 protein [100]. Subsequent studies proposed that BAP1 influences BRCA1 activity through direct binding to BRCA1-associated RING domain 1 (BARD1) and disrupts formation of the BRCA1–BARD1 complex, thus inhibiting BRCA1′s E3 ligase function [101]. However, a recent interactome study based on an affinity purification mass spectrometry approach showed that neither BRCA1 nor BARD1 was found associated with BAP1 [102]. Therefore, the association of BAP1 with BRCA1 and BARD1 is currently unclear. The tumor suppressor functions of BAP1 have been linked to its dual activity in the nucleus, where it has roles in DNA repair [80,81] and transcription [82,103,104,105], and regulation of cell death [83,106] and mitochondrial metabolism [107,108] in the cytoplasm. Being a tumor suppressor, loss of BAP1 through germline and somatic mutations has been directly linked to the predisposition of BAP1-mutated individuals to various malignancies including mesothelioma [109,110,111,112,113,114], uveal melanoma [115,116], cutaneous melanoma [117], and clear cell renal cell carcinoma [118,119,120]. Importantly, inherited BAP1 mutations in the form of truncating mutations have been detected in families with members diagnosed with mesothelioma, uveal melanoma, or breast cancer, suggesting a BAP1 familial cancer syndrome due to germline BAP1 mutations [109,121,122,123].

### 4.2. USP7

Identified in 1997, ubiquitin-specific protease 7 (USP7) was first characterized to interact with herpes simplex virus type 1 (HSV-1) immediate-early protein (Vmw110) of the HSV-1 regulatory protein and other viral proteins [124,125]. USP7 is the most widely studied DUB and considered to be an oncogene as it promotes tumor growth and negatively affects patient’s immune response to tumors [126,127]. Identified substrates and binding partners of USP7 are found to play crucial roles in immune response and tumor suppression, among others in a variety of human malignancies, which are discussed below.

In immune response, emerging data have shown on a functional link between USP7 and T regulatory cells (Treg) functions. Treg cells express the forkhead transcription factor Foxp3, which is important for Treg cell development [128]. Foxp3 is also shown to possess oncogenic functions including self-renewal and immunomodulation properties in tumor cells [84,129]. Foxp3 can be ubiquitinated at five different lysine residues (K249, K251, K263, K267, and K393) and can be stabilized by USP7 mediated deubiquitination, resulting in Treg number and function maintenance [130]. Aberrant USP7 overexpression reduces Foxp3 polyubiquitination and prevents its degradation. This, in turn, suppresses Treg function and promotes tumor growth [128]. Subsequent studies reveal that Foxp3 can be ubiquitinated and undergoes degradation by E3 ubiquitin ligase stress inducible protein 1 homology and U-Box containing protein 1 (STUB1). Further, heat shock protein 70 (Hsp70), STUB1, and Foxp3 form a complex, suggesting that these proteins bind and promote Foxp3 ubiquitination [85].

USP7 plays conflicting roles in p53 regulation through various mechanisms. p53 first interacts with the tumor necrosis factor (TNF) receptor-associated factor (TRAF) domain and C-terminal (amino acids 880–1050) of USP7, where, by acting as a tumor suppressor, USP7 then ubiquitinates p53 directly and prevents its degradation. Conversely, the TRAF domain and C-terminal (amino acids 801–1050) of USP7 can interact with MDM2 and increase its stability by removing ubiquitin from MDM2, an E3 ligase of p53, preventing its degradation [131]. MDM2 then ubiquitinates p53 and promotes its proteasomal degradation, thus leading to low p53 levels in tumor cells [86]. MDM2 is also shown to inhibit p53 transcription [64]. Further, disruption of MDM2 and p53 interaction induces p53 stabilization [132]. Crystallography and binding studies reveal that both MDM2 and p53 peptides bind to the same surface groove in USP7 [133].

### 4.3. USP22

Ubiquitin-specific protease 22 (USP22) is putated to be an oncogene. It is highly expressed in multiple malignancies and has been associated with poor overall survival (OS). Aberrant USP22 expression plays crucial roles in regulating DNA transcription, cell cycle transformation and maintaining genomic stability of tumors [87,134]. Apart from activating oncogenes such as BMI-1 and c-MYC, USP22 can inhibit the expression of tumor suppressors such as TP53 through ubiquitination, thus promoting proliferation of tumors [87]. USP22 is shown to regulate EGFR endocytosis through deubiquitination, resulting in sustained activation of the EGFR-dependent signaling pathway and promoting resistance to EGFR-TKIs in EGFR-mutated lung adenocarcinoma [88]. Further, USP22 is shown to facilitate cell-cycle progression and colorectal tumorigenesis by targeting CCNB1 while in glioblastoma, USP22 promotes tumorigenesis via stabilizing KDM1A [89,90]. Together, the evidence above clearly shows the roles of USP22 in driving tumor growth.

### 4.4. UCHL1

Ubiquitin C-terminal hydrolase L1 (UCHL1) belongs to the ubiquitin C-terminal hydrolases (UCHs) sub-family. Tissue UCHL1 distribution analysis shows that it is predominantly expressed in neuronal tissue, suggesting a role in the central nervous system [135]. UCHL1 overexpression is observed in some cancers including pancreatic, myeloma, prostate, osteosarcoma, and lung [136,137,138,139,140]. Further, UCHL1 expression positively correlates with chemotherapy resistance [91] and metastasis [141]. It has been suggested that UCHL1 is a tumor antigen capable of triggering a humoral immune response in lung cancer, thus pointing its potential as a biomarker in lung cancer [142].

### 4.5. Ataxin 3

Ataxin 3 (ATX3) belongs to the Josephin family of DUBs and it is mutated in spinocerebellar ataxia type 3 or Machado–Joseph disease [143]. ATX3 functions as a polyubiquitin chain-editing enzyme that controls protein folding and stability [144]. Apart from the Josephin domain, ATX3 contains other domains like the ubiquitin-interacting motif, suggesting that ATX3 is capable of interacting with two distal ubiquitins on a macromolecule [145]. ATX3 forms a complex with the p97/vasolin-containing protein (VCP) ubiquitin-dependent unfoldase/segregase and interacts with Ring finger protein 8 (RNF8) to form a physical and functional complex to regulate proteasome-dependent homeostasis [92]. RNF8 is rapidly recruited to DNA lesion sites during genotoxic stress where p97-ATX3 stimulates RNF8 removal from the chromatin to provide DNA repair pathway stability and promote cell survival after ionizing radiation (IR). Inactivation of the p97-ATX3 complex is further shown to sensitize cancer cells to IR. In testicular cancer (TC), ATX3 is overexpressed and correlates with tumor stages [93].

### 4.6. PSMD14

The regulatory particle non-ATPase 11 (PSMD14) is a member of the JAB1/MPN/Mov34 metalloenzyme (JAMM) family of metalloproteases, and is responsible for providing isopeptidase activities to reverse the effects of the ubiquitin-proteasome degradation system. PSMD14 plays a diverse role in various biological processes, including DNA repair, embryonic cell development, cell differentiation, programmed cell death and resistance to drugs [94,146,147]. Further, PSMD14 modulates tumor cell proliferation, through regulating the retinoblastoma protein (Rb) phosphorylation and cyclin-dependent kinases (CDKs) [95]. Upregulation of PSMD14, in hepatocellular carcinoma, has been associated with tumor promotion through E2F transcription factor 1 (E2F1) stabilization and its target genes [96].

### 4.7. A20

Tumor necrosis factor inducible protein A20 (A20) is a ubiquitin-editing enzyme that removes K63-linked ubiquitin chains from adaptor proteins, such as receptor-interacting serine/threonine-protein kinase 1 protein (RIPK1), and subsequently adds K48-linked polyubiquitin chains to trigger degradation. RIPK1 degradation triggers termination of nuclear factor Kappa light-chain enhancer of activated B (NF-κB) signaling in response to tumor necrosis factor (TNF), and prevents TNF-mediated apoptosis in a cell-dependent manner [148]. Though the cell death mechanism exerted by A20 is unclear, several mechanisms have been proposed. It has been suggested that A20 inhibits TNF-induced cell death by preventing interaction of RIPK1 and tumor necrosis factor receptor type 1-associated death domain protein (TRADD) to TNF receptor 1 (TNFR1), thereby inhibiting subsequent recruitment of FAS-associated death domain protein (FADD) and caspase-8 [149]. In a different study, A20 acts by impeding Cullin 3 (CUL3)-dependent ubiquitination and caspase-8 activation [97]. A20 dysregulation, in the form of promoter methylation and inactivating mutation, has been observed in lymphomas [150,151]. A20 expression can be regulated by specific miRNAs in a cellular context manner. MicroRNAs miR125a and miR125b can suppress A20 expression in diffuse large B-cell lymphoma, thus contributing to tumor cell proliferation through constitutive NF-κB signaling [98]. In contrast, miR29 is shown to protect A20 transcripts from degradation by preventing RNA-binding protein HuR binding and the subsequent recruitment of the RNA degradation complex in sarcoma cells [99].

## 5. Other Ubiquitin Modifiers

The E1-activating enzymes are a group of proteins also known as ubiquitin-like proteins (UBLs) as they share sequence homology and possess a similar three-dimensional structure to ubiquitin [152]. The eight human E1 enzymes identified are ubiquitin-like modifier activating enzyme 1 (UBA1), neural precursor cell expressed, developmentally downregulated 8 (NEDD8)-activating enzyme (NAE), small ubiquitin-like modifier (SUMO)-activating enzyme (SAE), UBA4, UBA5, UBA6, UBA7, and autophagy-related protein 7 (ATG7) [21,153,154]. E1s can be subdivided into canonical and noncanonical based on their structural and biochemical properties.

Among the eight E1 enzymes members identified, UBA1 has been shown to be most important where loss-of-function exerts the most unfavorable effect on the survival and growth of tumor cells [21,155]. In a genome-wide study to identify genes which are important for cancer survival through RNAi screening, UBA1 and UBA2 (encoding SAE active subunit) genes have been identified to be essential for survival after knockdown in large pan-cancer RNA interference screens in cancer cell lines [156]. Cancer cells can be identified according to stress phenotypes which include oxidative, metabolic, mitotic, and DNA damage stress [157]. Tumor cells rely on normal cellular machineries, such as the UPS system, to cope with these stresses. There is a tendency for tumor cells to rely on these machineries to a greater extent versus normal cells. This provides an avenue of therapeutic opportunities through stress overload and stress sensitization [157,158]. Therapeutic inhibition of E1 enzymes, UBA1 and NAE, induces tumor cell death through unfolded protein response (UPR) and proteotoxic stress in hematologic malignancies [159,160]. Neddylation of Cullin RING ligases (CRLs) by NAE enhances ubiquitination of CRL-bound protein substrates [161,162]. NAE inhibition has been shown to be a promising approach to treat cancers with the anti-E1 inhibitor, MLN4924. MLN4924 prevents CRL neddylation, leading to CRL substrate accumulation and DNA re-replication prior to tumor cell apoptosis [159]. Importantly, MLN4924 has been shown to reduce tumor growth in vivo. A single-nucleotide transition of alanine 171 to threonine of the NAE subunit ubiquitin-activating enzyme (UBA3), however, has been reported to reduce the enzyme’s affinity for MLN4924 and ATP while increasing NEDD8 activation at physiological ATP concentrations, which suggests a potential pitfall of this strategy [163].

## 6. UPS Inhibitors in Cancer Therapy

The progress in targeting the UPS has been slow and this delay has been attributed to the following reasons. First, most components of the ubiquitin system do not possess a well-defined catalytic pocket to allow binding of small inhibitors. Second, the ubiquitination process relies on the dynamic rearrangement of multiple protein–protein interactions that traditionally have been challenging to disrupt with small molecule inhibitors. Third, components of the UPS are shown to possess both oncogenic and tumor suppressor properties due to the complexity of their regulatory cellular processes. Despite these challenges, components of the UPS have been considered as attractive targets for cancer treatment. In the following sections, we introduce some inhibitors against components of the UPS that have been tested in preclinical and clinical studies (Table 3).

### 6.1. Targeting the Proteasome

Proteasome inhibitors were first developed for the treatment of cachexia in patients with advanced cancers. Cachexia is a condition characterized by a catabolic state of progressive wasting [69]. It was hypothesized that a proteasome inhibitor could prevent protein degradation and muscle wasting [164]. MG132, a peptide aldehyde, is a well-known potent proteasome inhibitor commonly used in research. Though MG132 has been proven to be a valuable research tool, it has never been tested clinically due to its rapid oxidation [165].

Bortezomib (velcade), a peptide boronate, was the first U.S. Food and Drug Administration (FDA)-approved proteasome inhibitor. It can reversibly form tetrahedral adducts with Thr1 residues in the catalytic β5 subunits of 19S, and exhibits phenomenal potency in vitro. Bortezomib initially showed only unremarkable activity in solid cancer patients in phase I trials. Subsequently, it was reported to promote disease regression in multiple myeloma patients [166] through stabilization of I-κB, thus suppressing NF-κB signaling [167]. Further, bortezomib induces accumulation of two negative regulators of the cell cycle, p27KIP1 and p53 [168]. Additional anticancer properties of bortezomib are that it can induce pro-apoptotic protein BAX and oxidative stress which tilt the balance towards apoptosis [169,170]. However, bortezomib exhibits dose-limiting toxicities and multiple side effects have been reported including peripheral neuropathy, myelosuppression, and cardiotoxicity. Approximately 60% of patients treated with bortezomib developed resistance to this drug, and the FDA approved the use of bortezomib, in combination with chemotherapeutic agents such as doxorubicin and dexamethasone, to overcome resistance in clinical trials [171,172].

Carfilzomib (kyprolis), a tetrapeptide epoxyketone, is the second proteasome inhibitor approved by the FDA [76]. Carfilzomib binds to both hydroxyl and the free α-amino group of Thr1 in the catalytic β5 subunits and blocks the binding of substrate proteins to β5 [173,174,175]. Carfilzomib can irreversibly inhibit proteasomal activity to less than 20%, and has been shown to induce antitumor response in bortezomib-resistant multiple myeloma [175]. It possesses a more selective mechanism of action and exhibits high chemical stability, which is shown by fewer side effects when compared to bortezomib [176,177].

Ixazomib (Ninlaro) was developed as a second-generation proteasome inhibitor and the first one FDA-approved, as an oral administered drug, for multiple myeloma [178]. Similar to the first generation, ixazomib triggers apoptotic cell death in multiple myeloma cell lines and enhances the expression of pro-apoptotic genes [178]. Unlike bortezomib, ixazomib does not affect the mitochondrial serine protease Htr/Omi, which is a neuropathy-related off-target of bortezomib [172]. Other proteasome inhibitors currently undergoing clinical trials include delanzomib, marizomib, and oprozomib (Table 3).

### 6.2. Targeting Ubiquitinases

MLN4924 inhibits the NEDD8-activating enzyme (NAE) E1 enzyme where it forms an adduct with NAE and prevents Cullin-RING ligases (CRLs) neddylation [159,179]. Blocking CRL neddylation restricts tumor cell growth, resulting in elevated substrate levels, which then triggers cell death through DNA damage response, autophagy, cell-cycle arrest, and apoptosis [180]. MLN4924 has shown clinical activity in a phase I clinical trial of acute myelogenous leukemia (AML) [181]. It is also shown to inhibit angiogenesis during tumor development [182]. TAK981 (SAE) and TAS4464 (NAE) are two other E1 inhibitors currently undergoing clinical trials against various cancers (Table 3).

**Table 3:** Summary of UPS inhibitors which are FDA-approved and/or tested in clinical trials described in this review.

Inhibitor	Target	Cancer Type	Clinical Trial	Reference
*Bortezomib*	Proteasomal inhibitor	Multiple myeloma, Mantle cell lymphoma, Leukemia, Neuroblastoma, Head and Neck, Thyroid, Hepatocellular	FDA approved	www.clinicaltrials.gov [126,166,167,169,170,171,172,177]
*Carfilzomib*	Proteasomal inhibitor	Multiple myeloma, Lymphoma, Relapsed and/or refractory multiple myeloma, Leukemia, Lung, Thyroid, Refractory renal cell carcinoma	FDA approved	www.clinicaltrials.gov [54,173,174,175,176,177]
*Ixazomib*	Proteasomal inhibitor	Multiple myeloma, Relapsed and/or refractory multiple myeloma, Lymphoma, Leukemia, Breast, Glioblastoma, Renal cell carcinoma, Hodgkin and T cell lymphoma	FDA approved	www.clinicaltrials.gov [178]
*Delanzomib*	Proteasomal inhibitor	Non-Hodgkin’s lymphoma	Phase I	www.clinicaltrials.gov
*Marizomib*	Proteasomal inhibitor	Multiple myeloma, Advanced solid tumors	Phase I/II	www.clinicaltrials.gov
*Oprozomib*	Proteasomal inhibitor	Multiple myeloma, Glioma, Pancreatic, Lung, Melanoma, Lymphoma, Glipblastoma	Phase I/II/III	www.clinicaltrials.gov
*MLN4924*	NAE and UBA1(E1)	Advanced malignant solid tumors, Melanoma, Hepatocellular, B cell lymphoma, Hematologic malignancies, Acute myelocytic leukemia	Phase I/II/III	www.clinicaltrials.gov
*TAK981*	SAE (E1)	B cell lymphoma, colorectal, non-Hodgkin’s, Advcnced/metasiatic solid tumors	Phase I/II	www.clinicaltrials.gov
*TAS4464*	NAE (E1)	Multiple myeloma, non-Hodgkin lymphoma	Phase I/II	www.clinicaltrials.gov
*SAR-405838*	MDM2 (E2)	Solid tumors	Phase I	www.clinicaltrials.gov [172,183]
*CGM-097*	MDM2 (E2)	Advanced p53 wildtype solid tumors	Phase I	www.clinicaltrials.gov [184,185]
*DS-3032b*	MDM2 (E2)	Acute myelocytic leukemia	Phase I/II	www.clinicaltrials.gov [186,187]
*Debio1143 (AT-406)*	cIAP1/2 (E3)	Acute myeloid leukemia	Phase I	www.clinicaltrials.gov [188]
*LC-161*	IAP (E3)	Advanced solid tumors	Phase I	www.clinicaltrials.gov [189]
*Birinapant*	IAP (E3)	Solid tumors	Phase I/II	www.clinicaltrials.gov [190]
*Pimozide*	USP1	Glioma, Non-small cell lung cancer	FDA approced for Tourette’s syndrome; Preclinical	[191,192]
*Mitoxantrone*	USP11	Metastatic crastrate -resistant prostate, Acute myeloid leukemia, Advanced breast cancer, non-Hodgkin’s lymphoma, Primary liver	FDA approved	[193,194,195,196,197,198,199,200,201]

Nutlin-3a is the first small molecule inhibitor of the oncogene MDM2, a negative regulator of p53, and this prevents interaction between MDM2 and p53 [132]. RO-5503781 (idasanutlin), a Nutlin-3a derivative, is designed and developed as an oral inhibitor for the treatment of various malignancies including glioblastoma, diffuse large B cell lymphoma, multiple myeloma, prostate, and AML in preclinical studies [172,183]. However, RO-5503781 was terminated in phase II clinical trials. Similar to RO-5503781, Nutlin-3a-derivative MK-8242 was terminated in phase I clinical trials for advanced solid tumors and recurrent AML and liposarcomas. Other Nutlin-3a derivatives such as SAR-405838, CGM-097, and DS-3032b are currently in phase I clinical trials for patients with advanced solid tumors and lymphoma, either alone or in combination with chemotherapy [184,185,186,187,202].

The inhibitors of apoptosis (IAP) proteins are ubiquitin E3 ligases, responsible for cell survival and oncogenesis. cIAP1 and cIAP2 paralogs contain three N-terminal baculoviral IAP repeat (BIR) domains and a C-terminal E3 ligase really interesting new gene (RING) domain. IAP antagonist compounds, known as second mitochondria-derived activator of caspase (SMAC) mimetics, bind to BIR domains and activate RING-dependent autoubiquitylation which leads to IAP destruction [203]. Debio1143 (AT-406) is a small molecule SMAC mimetic IAP antagonist that has entered clinical development. Debio1143 is reported to inhibit the cellular inhibitor of apoptosis 1 (cIAP-1) in acute myeloid leukemia where about a third of patients enrolled achieved complete remission within the study period [188]. Elevated plasma TNFα and IL-8 are associated with responders and patients showed good tolerability in combination with chemotherapy. Other SMAC mimetic IAP antagonists such as LCL161 and birinapant have entered phase II trials [189,190,204]. Debio1143 and birinapant can preferentially target cIAP1 and cIAP2 rather than X-linked inhibitor of apoptosis protein (XIAP), while LCL161 is a pan-IAP inhibitor that possesses similar affinity to XIAP, cIAP1, and cIAP2 [205,206,207,208]. Hence, the potency of SMAC mimetics relies on their specificity to IAPs. LCL161 and birinapant are among the most commonly used SMAC mimetics due their therapeutic effectiveness and known mechanism of actions demonstrated in both preclinical and clinical studies.

### 6.3. Targeting DUBs

Proteasome-associated DUBs are responsible for deubiquitination of target proteins. Many deubiquitinases are dysregulated and their roles in cancer rely on the substrates they deubiquitinate. Although potent irreversible inhibitors of DUBs such as ubiquitin aldehyde or ubiquitin vinyl sulfone have been widely used as research tools, only a few DUB inhibitors have entered clinical trials for certain cancers. The development of selective DUB inhibitors has, however, been hampered by limited knowledge of DUB biology and the pleiotropic properties of small-molecule DUB inhibitors.

Pimozide was developed as an antipsychotic drug and approved for the treatment of Tourette’s syndrome by the FDA. It is a reversible inhibitor of the enzymatic ubiquitin-specific protease 1/ USP1-associated factor 1 (USP1/UAF1) complex activity. The USP1/UAF1 complex plays an important role in the regulation of homologous recombination (HR)-mediated double-strand break (DSB) repair [209]. In cisplatin-resistant NSCLC cells, pimozide inhibits USP1/UAF1, and acts synergistically with cisplatin to inhibit cell proliferation [191]. Further, pimozide is shown to resensitize glioma tumors to radiation therapy [192].

Mitoxantrone (novantrone) was approved by the FDA for use in metastatic castrate-resistant prostate cancer [193]. Mitoxantrone inhibits USP11 catalytic activity and impairs the DNA-damage repair (DDR) system in pancreatic ductal adenocarcinoma (PDA) in vitro [210]. USP 11 is a tumor suppressor where it is shown to interact with ubiquitinated BRCA2 and exerts a prosurvival function in response to cellular DNA damage [211]. Further, it is reported to inhibit transforming growth factor (TGF)-β1-induced phosphorylation of SMAD2/3 and type I TGF-β receptor by destabilizing the type II TGF-β receptor in lung fibroblasts [212]. Mitoxantrone has shown clinical activity in relapsed/refractory acute myeloid leukemia either administered alone or in combination with chemotherapy [194,195]. Other cancer types which show response and are well-tolerated to mitoxantrone include advanced breast cancer, non-Hodgkin’s lymphoma, and primary liver cancer [196,197,198,199,200,201].

P5091 and b-AP15 are two other DUB inhibitors which have shown promise in preclinical studies. P5091 is shown to selectively inhibit USP7 in multiple myeloma both in vitro and in vivo [213]. USP7 plays a crucial role in p53 regulation, and P5091 shows weaker activity after USP7 inhibition, thus demonstrating its specificity. Importantly, P5091 induces apoptotic cell death in patient multiple myeloma cells resistance to bortezomib, lenalidomide, and dexamethasone [213]. Nevertheless, P5091 shares the same limitation as Nutlins as they only stabilize wild-type p53, while the majority of tumors are mutated p53. b-AP15 inhibits both UCH37 and USP14, and induces accumulation of ubiquitinated substrates [214]. B-AP-15 demonstrates outstanding efficacy in solid tumor and acute myeloid leukemia models in vivo.

## 7. Conclusions

Frequent aberrant UPS activity seen in human malignancies indicates that the proteasome and components of the UPS are attractive therapeutic targets. Targeting the proteasome, in the clinic, has achieved success with FDA-approved proteasome inhibitors such as bortezomib, carfilzomib, and ixazomib. Being the last step in the UPS, the use of proteasome inhibitors has shown undesirable side effects arising from the action of upstream UPS components. This shows that there is an untapped potential for the development of drugs against other components of the UPS. Thus, ubiquitin-activating steps, E2, E3, and DUBs can be exploited for inhibition [215,216]. Unfortunately, most of these inhibitors show good efficacy in culture models but less so in animal models and clinical trials [217,218,219]. Traditionally, the ubiquitin activating steps and degradation possess the greatest potential due to presence of well-defined activity pockets but face issues of substrate specificity. The other UPS components, however, do not possess defined pockets for targeting with small inhibitors. Hence, delay in the development of successful UPS inhibitors can be attributed to the lack of knowledge of target protein structures and identifiable activity pockets for inhibitor binding. Advances in technology such as computer-aided design, mass spectrophotometry, and high throughput screening may aid in the identification of suitable candidates. Further, the occurrence of oncogenic signaling together with aberrant UPS activity may affect the success of future UPS inhibitors. Nevertheless, a greater effort is required to elucidate the functions of aberrant UPS at both preclinical and clinical levels to better understand their roles in human malignancies to develop alternative paradigms for therapeutic intervention.

## Data Availability

Not applicable.
